# Supplementary material for: Bacterial PncA improves diet-induced NAFLD in mice by enabling the transition from nicotinamide to nicotinic acid
Source: Commun Biol. 2023 Mar 2;6:235. doi: 10.1038/s42003-023-04613-8 (PMC9981684; doi:10.1038/s42003-023-04613-8)
Supplement: Supplementary file 2 — Supplementary Information-New [file 42003_2023_4613_MOESM2_ESM.pdf]

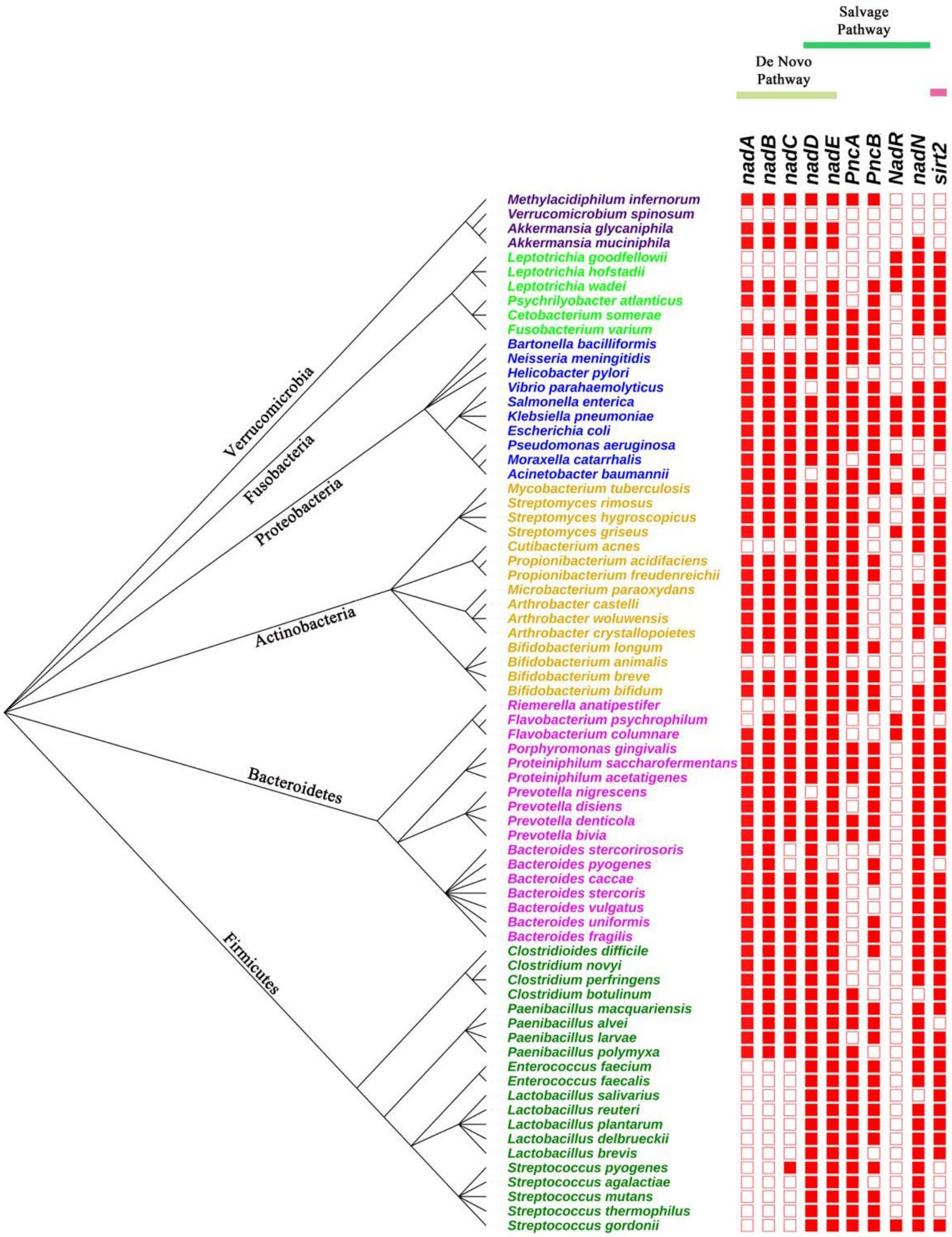

Supplementary Figure 1. Genes associated with NAD$^+$ synthesis in different taxa of bacteria

nadA, *nadB*, and *nadC* genes are involved in the NAD$^+$ *de novo* synthesis pathway, and *nadD* and *nadE* genes are involved in the *de novo* and salvage pathways. *PncA*, *PncB*, *nadV*, *nadR* and *nadN* genes are only involved in the NAD$^+$ synthesis pathway using precursors. The solid red box indicates that the species has the corresponding gene, while the blank red box indicates that they do not have that gene. Bacteria in different phyla are represented by different colors in the evolutionary tree.

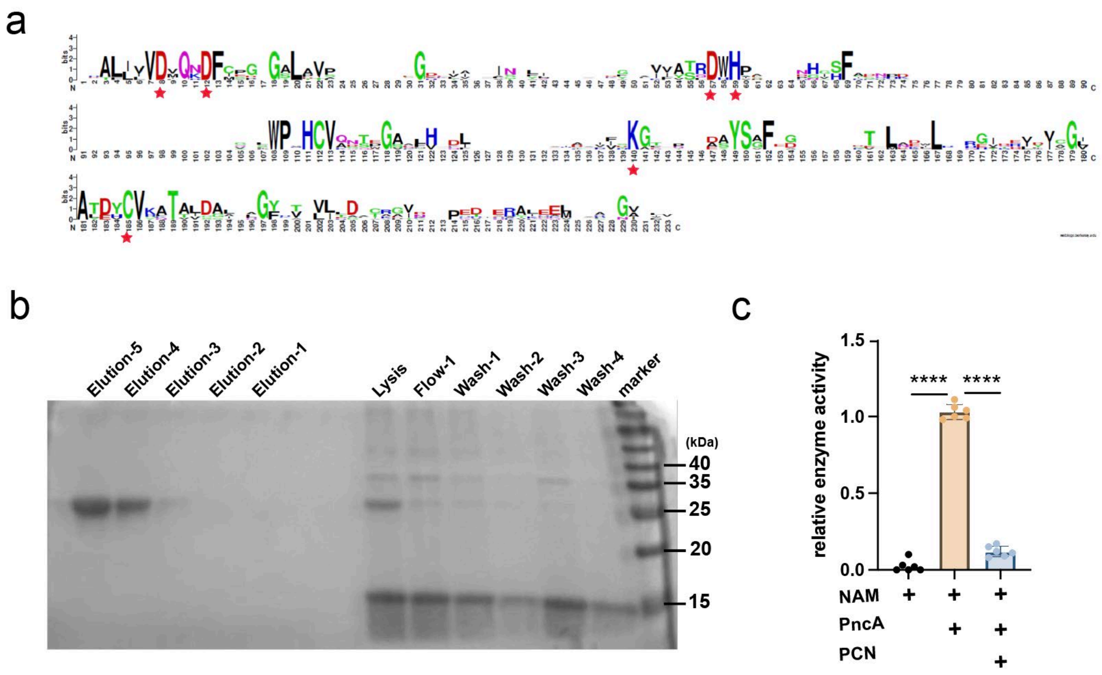

Supplementary Figure 2. PCN inhibits PncA activity *in vitro*.

a PncA sequence alignment drawn by weblogo [51]. The key amino acids involved in catalysis are labeled by red stars. **b** Coomassie blue staining of purified PncA. **c** Enzyme activity of *Escherichia coli* PncA *in vitro* (n=6,****p<0.0001).

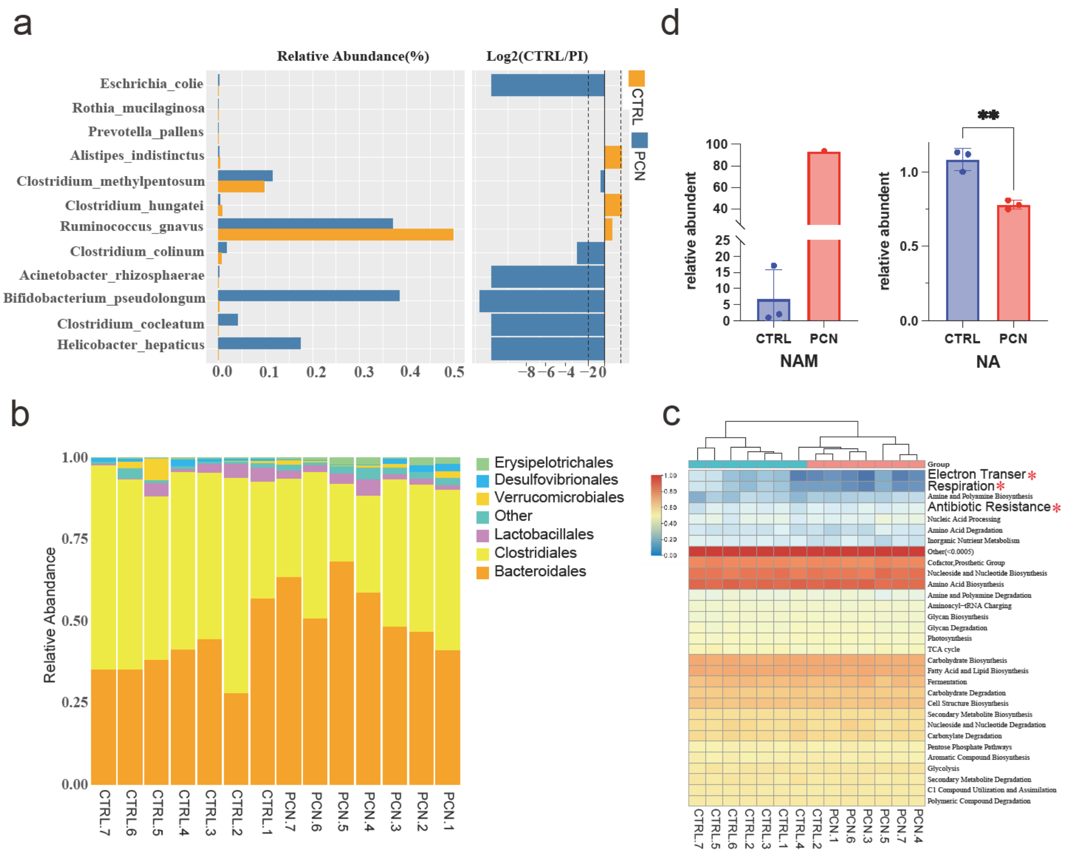

Supplementary Figure 3. Effects of PCN on the intestinal microbiota.

a Distribution of bacteria at the species level. **b** Distribution of bacteria at the order level. **c** KEGG pathways of bacteria enriched after PCN treatment. **d** Abundance of NAM and NA in feces of mice (n=3, however NAM was not detected in two samples of PCN group).

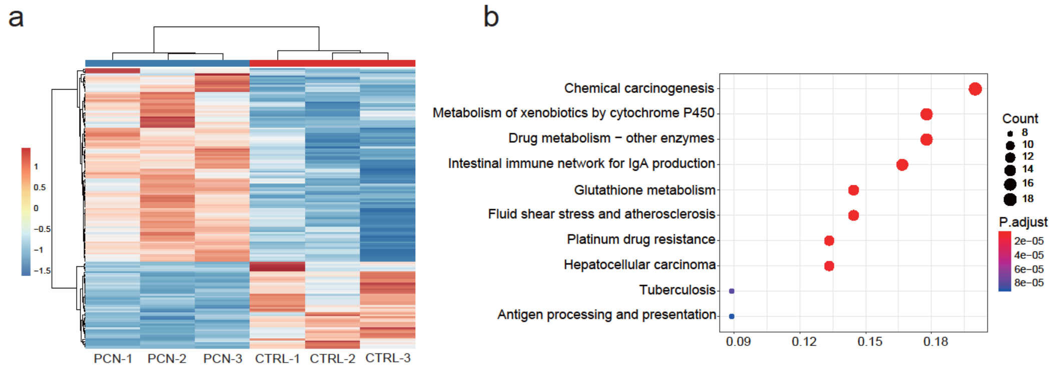

Supplementary Figure 4. Effects of PCN on host.

a Heatmap of differentially expressed genes in the liver in the PCN group and control group. **b** KEGG enrichment analysis of differentially expressed genes.

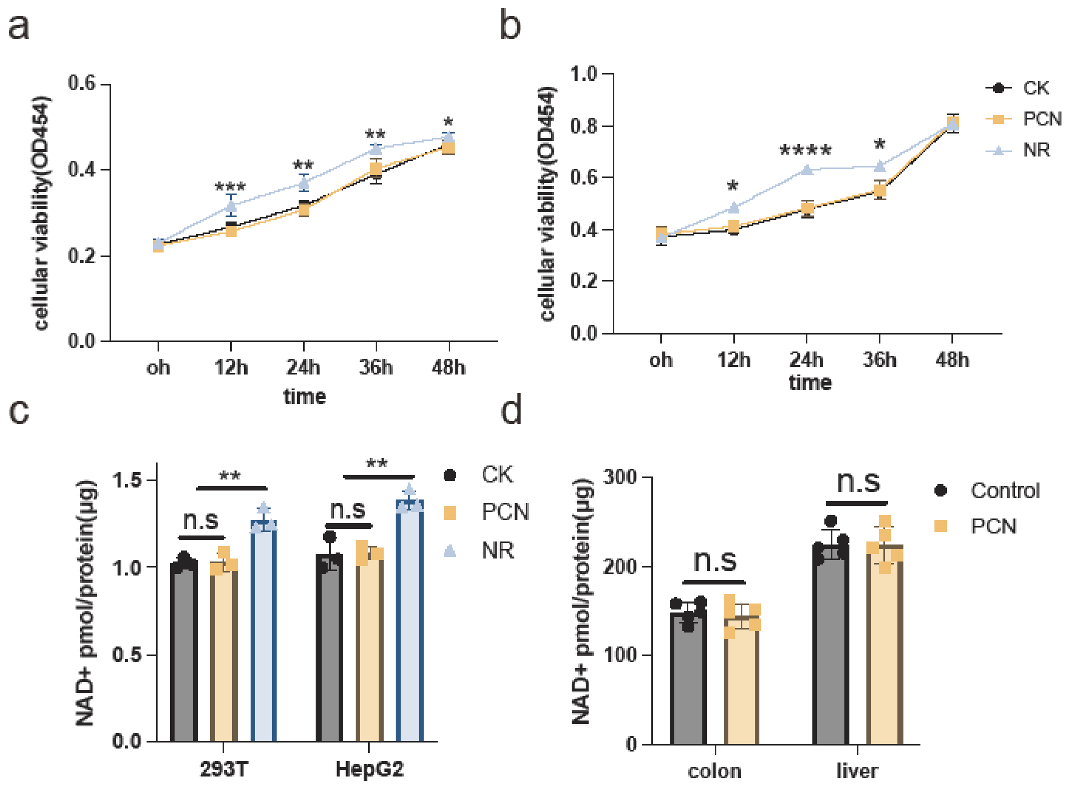

Supplementary Figure 5. PCN does not affect the growth status and NAD$^+$ level of human cells as well as antibiotic-treated mouse colon and liver.
a Cell vitality of 293T cells after NR and PCN treatment (n=3, *p<0.05, **p<0.01, ***p<0.001). **b** Cell vitality of HepG2 cells after NR and PCN treatment (n=3, *p<0.05, ****p<0.0001). **c** NAD+ level in 293T and HepG2 cells after NR and PCN treatment (n=3, **p<0.01, n.s=no significance). **d** NAD+ level in antibiotic-treated mouse colon and liver (n=5, n.s=no significance).

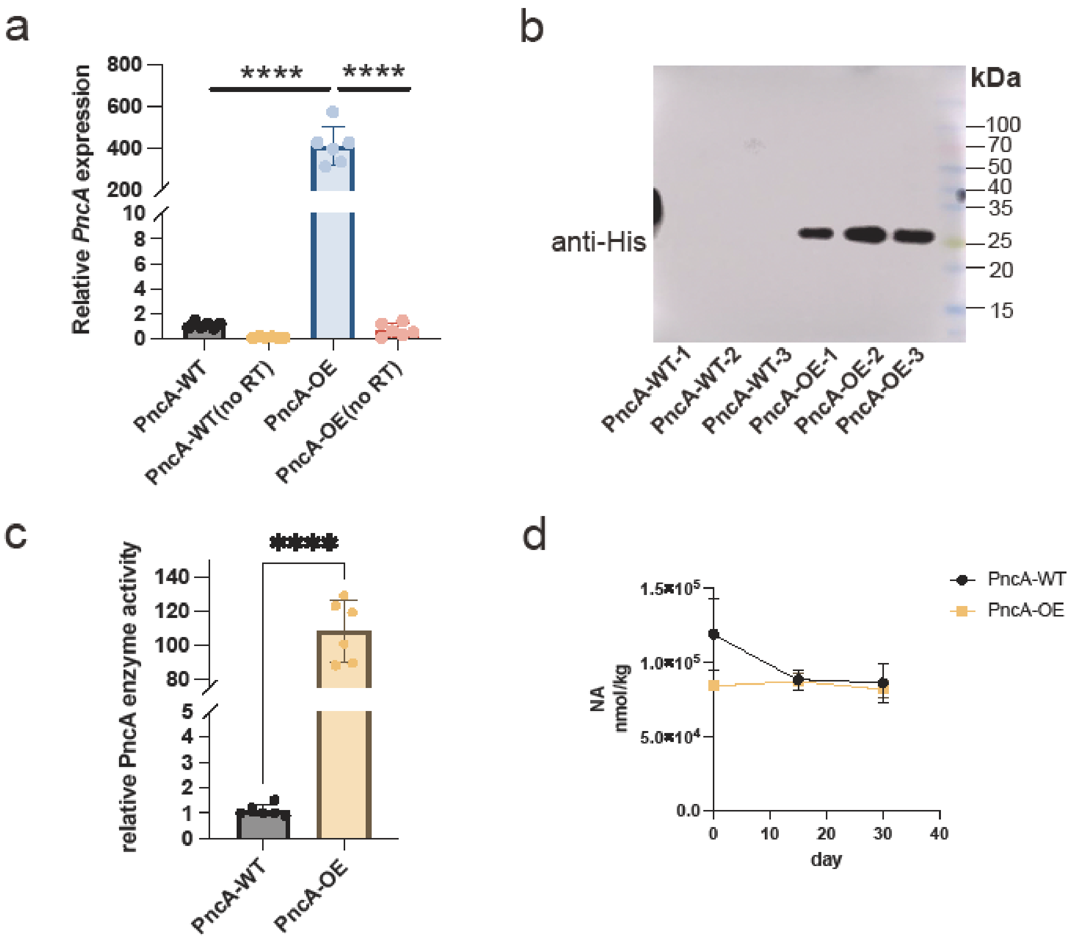

Supplementary Figure 6. Construction and validation of PncA overexpression *Escherichia coli in vitro* and *in vivo*.

a Relative gene expression of *PncA* in WT and PncA-OE *E.coli* (n=6, ****p<0.0001). **b** Western blotting of His-PncA in WT and PncA-OE *E. coli*. **c** Enzyme activity assay of *E. coli* lysis. (n=6, ****p<0.0001, n.s=no significance). **d** Abundance of NA in mouse feces after supplementation with *E. coli* (n=3).

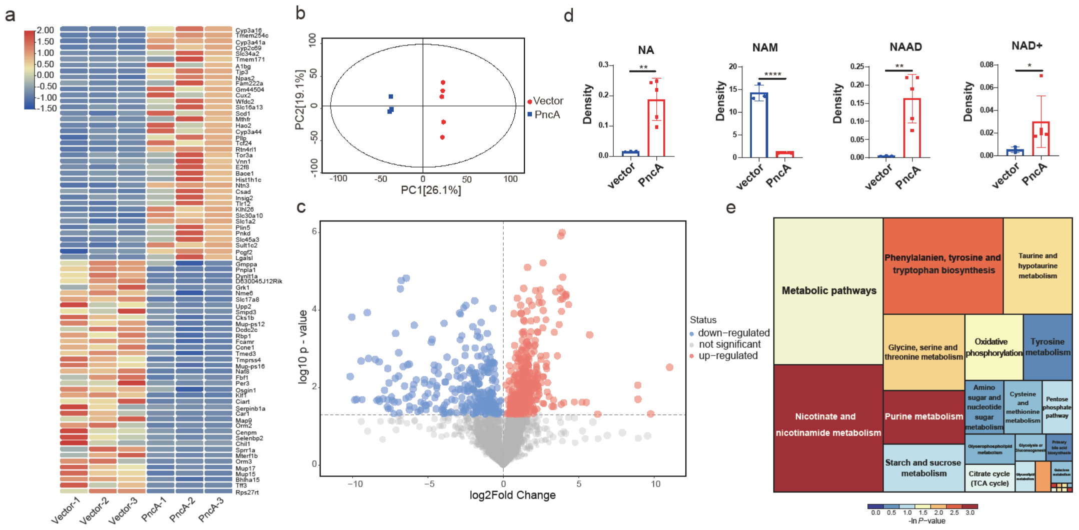

Supplementary Figure 7. PncA affected the genes and metabolites related to lipid metabolism in NAFLD model mice

a Heatmap of differentially expressed genes between the PncA and vector groups. **b** PCA of metabolites in PncA and vector groups. **c** Volcano plot of metabolites in PncA and vector groups. The colored dots represent differentially regulated metabolites between the two groups. **d** Relative contents of NA, NAM, NAAD and NAD+ in mouse liver (n=3 in vector group, n=5 in PncA group, *p<0.05, **p<0.01, ****p<0.0001). **e** Enriched KEGG pathways of differentially regulated metabolites.

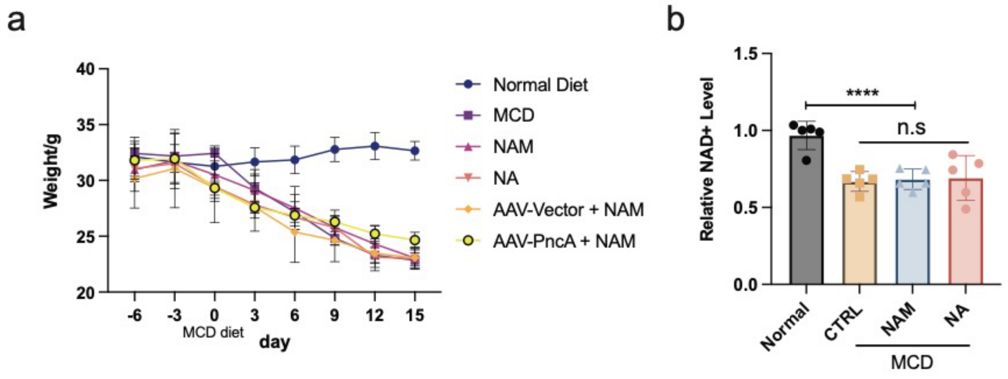

Supplementary Figure 8. NA did not increase the NAD$^+$ level in NAFLD mice

a Body weight of mice during the experiment after supplementation with the MCD diet. The body weight gradually decreased. **b** Relative NAD+ level of liver in the normal diet group and MCD diet group treated with PBS, NAM or NA. (n=5, ****p<0.0001, n.s=no significance)
