# Supplementary material for: Bacterial PncA improves diet-induced NAFLD in mice by enabling the transition from nicotinamide to nicotinic acid
Source: Commun Biol. 2023 Mar 2;6:235. doi: 10.1038/s42003-023-04613-8 (PMC9981684; doi:10.1038/s42003-023-04613-8)
Supplement: Supplementary file 3 — Description of Additional Supplementary Files [file 42003_2023_4613_MOESM3_ESM.pdf]

Description of Additional Supplementary Files

File name: Supplementary Data 1

Description: The raw metabolome analysis data of liver after PncA overexpression.

File Name: Supplementary Data 2

Description: The source data for Fig. 2a, 2c-f, 3d, 4a, 4c-e, 5a-d, S2c, S3d, S5a-d, S6a, S6c-d, S7c, S8a-b behind the graphs in the paper.
