# Supplementary material for: Bacterial PncA improves diet-induced NAFLD in mice by enabling the transition from nicotinamide to nicotinic acid
Source: Commun Biol. 2023 Mar 2;6:235. doi: 10.1038/s42003-023-04613-8 (PMC9981684; doi:10.1038/s42003-023-04613-8)
Supplement: Supplementary file 6 — Reporting summary [file 42003_2023_4613_MOESM6_ESM.pdf]

nature portfolio

Corresponding author(s): Hailiang Liu

Last updated by author(s): Feb 6, 2023

Reporting Summary

Nature Portfolio wishes to improve the reproducibility of the work that we publish. This form provides structure for consistency and transparency in reporting. For further information on Nature Portfolio policies, see our Editorial Policies and the Editorial Policy Checklist.

Statistics

For all statistical analyses, confirm that the following items are present in the figure legend, table legend, main text, or Methods section.

n/a	Confirmed	
☐	☒	The exact sample size (*n*) for each experimental group/condition, given as a discrete number and unit of measurement
☐	☒	A statement on whether measurements were taken from distinct samples or whether the same sample was measured repeatedly
☐	☒	The statistical test(s) used AND whether they are one- or two-sided<br>*Only common tests should be described solely by name; describe more complex techniques in the Methods section.*
☒	☐	A description of all covariates tested
☒	☐	A description of any assumptions or corrections, such as tests of normality and adjustment for multiple comparisons
☐	☒	A full description of the statistical parameters including central tendency (e.g. means) or other basic estimates (e.g. regression coefficient) AND variation (e.g. standard deviation) or associated estimates of uncertainty (e.g. confidence intervals)
☐	☒	For null hypothesis testing, the test statistic (e.g. *F*, *t*, *r*) with confidence intervals, effect sizes, degrees of freedom and *P* value noted<br>*Give P values as exact values whenever suitable.*
☒	☐	For Bayesian analysis, information on the choice of priors and Markov chain Monte Carlo settings
☒	☐	For hierarchical and complex designs, identification of the appropriate level for tests and full reporting of outcomes
☐	☒	Estimates of effect sizes (e.g. Cohen's *d*, Pearson's *r*), indicating how they were calculated

Our web collection on statistics for biologists contains articles on many of the points above.

Software and code

Policy information about availability of computer code

Data collection	no software was used.
Data analysis	GraphPad Prism 8, R software (v3.4.1), Adobe Photoshop, Adobe Illustrator, Python (version 2.7)<br>Readfq v8, FLASH v1, USEARCH v7, motor v1, QIIME, ggplot2, SOAPnuke v1, HISAT2 v2, Bowtie2 v2

For manuscripts utilizing custom algorithms or software that are central to the research but not yet described in published literature, software must be made available to editors and reviewers. We strongly encourage code deposition in a community repository (e.g. GitHub). See the Nature Portfolio guidelines for submitting code & software for further information.

Data

Policy information about availability of data

All manuscripts must include a data availability statement. This statement should provide the following information, where applicable:

- Accession codes, unique identifiers, or web links for publicly available datasets
- A description of any restrictions on data availability
- For clinical datasets or third party data, please ensure that the statement adheres to our policy

The RNA-seq raw sequencing data files generated in this study are available in the NCBI's Sequence Read Archive (SRA) under BioProject accession number PRJNA780659. The original 16S rRNA sequence data are available at the NCBI by accession number PRJNA780391.

Human research participants

Policy information about studies involving human research participants and Sex and Gender in Research.

Reporting on sex and gender	*Use the terms sex (biological attribute) and gender (shaped by social and cultural circumstances) carefully in order to avoid confusing both terms. Indicate if findings apply to only one sex or gender; describe whether sex and gender were considered in study design whether sex and/or gender was determined based on self-reporting or assigned and methods used. Provide in the source data disaggregated sex and gender data where this information has been collected, and consent has been obtained for sharing of individual-level data; provide overall numbers in this Reporting Summary. Please state if this information has not been collected. Report sex- and gender-based analyses where performed, justify reasons for lack of sex- and gender-based analysis.*
Population characteristics	*Describe the covariate-relevant population characteristics of the human research participants (e.g. age, genotypic information, past and current diagnosis and treatment categories). If you filled out the behavioural & social sciences study design questions and have nothing to add here, write "See above."*
Recruitment	*Describe how participants were recruited. Outline any potential self-selection bias or other biases that may be present and how these are likely to impact results.*
Ethics oversight	*Identify the organization(s) that approved the study protocol.*

Note that full information on the approval of the study protocol must also be provided in the manuscript.

Field-specific reporting

Please select the one below that is the best fit for your research. If you are not sure, read the appropriate sections before making your selection.

☒ Life sciences ☐ Behavioural & social sciences ☐ Ecological, evolutionary & environmental sciences

For a reference copy of the document with all sections, see nature.com/documents/nr-reporting-summary-flat.pdf

Life sciences study design

All studies must disclose on these points even when the disclosure is negative.

Sample size	sample size were determined by our previous research experiences. For most experiments, 6 replications could produce stable and repeatable results. As for high throughput sequencing, 3 replications is a more ideal choice considering the repeatability and the cost.
Data exclusions	No data were excluded from analysis
Replication	All replication attempts were successful. However, for high throughput sequencing, considering the cost and time needed, we didn't repeat those experiments.
Randomization	C57 mice were purchased from company and be randomly assigned to different experiment group. Mouse liver sections used for imaging were selected randomly.
Blinding	*Describe whether the investigators were blinded to group allocation during data collection and/or analysis. If blinding was not possible, describe why OR explain why blinding was not relevant to your study.*

Reporting for specific materials, systems and methods

We require information from authors about some types of materials, experimental systems and methods used in many studies. Here, indicate whether each material, system or method listed is relevant to your study. If you are not sure if a list item applies to your research, read the appropriate section before selecting a response.

Materials & experimental systems

n/a	Involved in the study
☐	☒ Antibodies
☐	☒ Eukaryotic cell lines
☒	☐ Palaeontology and archaeology
☐	☒ Animals and other organisms
☒	☐ Clinical data
☒	☐ Dual use research of concern

Methods

n/a	Involved in the study
☒	☐ ChIP-seq
☒	☐ Flow cytometry
☒	☐ MRI-based neuroimaging

Antibodies

Antibodies used	Anti-his (M20001; Aamart, Shanghai, China)
Validation	Anti-his has been validated for use in immunohistochemistry, western blotting and ELISA as stated on the Abmart product page.

Eukaryotic cell lines

Policy information about cell lines and Sex and Gender in Research

Cell line source(s)	HEK293T cells (human, ATCC)<br>HepG2 cells (human, ATCC)
Authentication	None of the cell lines have been authenticated.
Mycoplasma contamination	The mycoplasma contamination status of all cultures was monitored monthly by PCR. No mycoplasma contaimination was detected in the cell culture.
Commonly misidentified lines<br>(See ICLAC register)	No commonly misidentified cell lines were used.

Animals and other research organisms

Policy information about studies involving animals; ARRIVE guidelines recommended for reporting animal research, and Sex and Gender in Research

Laboratory animals	Wild type C57BL/6J
Wild animals	No wild animals were used in this study.
Reporting on sex	Only female mice were used in our research.
Field-collected samples	No field-collected samples were used in this study.
Ethics oversight	All experiments were carried out following the national guidelines for the housing and care of laboratory animals (Ministry of Health, China), and the protocol complied with institutional regulations after review and approval by the Institutional Animal Ethics Committee at the Laboratory Animal Research Center, Tongji University (TJAA09220102).

Note that full information on the approval of the study protocol must also be provided in the manuscript.
